# Therapeutic immunomodulation by rationally designed nucleic acids and nucleic acid nanoparticles

**DOI:** 10.3389/fimmu.2023.1053550

**Published:** 2023-01-31

**Authors:** Martin Panigaj, Elizabeth Skelly, Damian Beasock, Ian Marriott, M. Brittany Johnson, Jacqueline Salotti, Kirill A. Afonin

**Affiliations:** ^1^ Nanoscale Science Program, Department of Chemistry, The University of North Carolina at Charlotte, Charlotte, NC, United States; ^2^ Institute of Biology & Ecology, Faculty of Science, Pavol Jozef Safarik University in Kosice, Kosice, Slovakia; ^3^ Department of Biological Sciences, University of North Carolina at Charlotte, Charlotte, NC, United States; ^4^ Mouse Cancer Genetics Program, Center for Cancer Research, National Cancer Institute, Frederick, MD, United States

**Keywords:** PRR, PAMP, DAMP, NANPs, immunomodulation, innate immune system, therapy

## Abstract

The immune system has evolved to defend organisms against exogenous threats such as viruses, bacteria, fungi, and parasites by distinguishing between “self” and “non-self”. In addition, it guards us against other diseases, such as cancer, by detecting and responding to transformed and senescent cells. However, for survival and propagation, the altered cells and invading pathogens often employ a wide range of mechanisms to avoid, inhibit, or manipulate the immunorecognition. As such, the development of new modes of therapeutic intervention to augment protective and prevent harmful immune responses is desirable. Nucleic acids are biopolymers essential for all forms of life and, therefore, delineating the complex defensive mechanisms developed against non-self nucleic acids can offer an exciting avenue for future biomedicine. Nucleic acid technologies have already established numerous approaches in therapy and biotechnology; recently, rationally designed nucleic acids nanoparticles (NANPs) with regulated physiochemical properties and biological activities has expanded our repertoire of therapeutic options. When compared to conventional therapeutic nucleic acids (TNAs), NANP technologies can be rendered more beneficial for synchronized delivery of multiple TNAs with defined stabilities, immunological profiles, and therapeutic functions. This review highlights several recent advances and possible future directions of TNA and NANP technologies that are under development for controlled immunomodulation.

## Immunorecognition of nucleic acids

From prokaryotes to eukaryotes, all cellular forms of life possess a variety of conserved defense mechanisms against pathogens. Bacteria and archaea have evolved multiple intracellular immune systems to protect against viral phage infections, including restricted-modification (R-M), prokaryotic Argonaute proteins (pAgo), clustered regularly interspaced palindromic repeats (CRISPR) and CRISPR associated (Cas) proteins, abortive infection (Abi) and the more recently discovered antiviral STAND NTPase (Avs) homolog proteins (1, 2).

Conceptually parallel to eukaryotic organisms, prokaryotes have both innate (e.g., R-M and pAgo) and adaptive (e.g., CRISPR/Cas) systems; most of which target invading nucleic acids (1, 3). The R-M and similar systems are based on the endonuclease-mediated cleavage of any DNA that lacks specific epigenetic modifications. CRISPR/Cas-mediated immunological memory consists of the insertion of short DNA sequences from intruding DNA into CRISPR arrays in the host genome, ultimately providing sequence-specific cleavage/degradation of foreign nucleic acids after a second encounter (4, 5).

In eukaryotes, defense against pathogenic infection involves multiple cellular and molecular strategies. One example of protection against pathogenic nucleic acids is RNA interference (RNAi), which is conserved from unicellular eukaryotes to mammals. RNAi machinery has many functions, including the recognition of ‘‘non-self’’ double-stranded RNAs originated from viruses and retrotransposons triggering silencing of the target RNA (6). Small silencing RNAs include small interfering RNAs (siRNAs), microRNAs (miRNAs), and PIWI-interacting RNAs (piRNAs) that regulate not only antimicrobial immunity but also “self” gene expression. In cases of viral infection, Dicer-dependent production of virus-derived small interfering RNAs (vsiRNAs) or Dicer-independent production of virus-derived piRNAs (vpiRNAs) can guide specific virus elimination (7).

Metazoan somatic cells have evolved cell-autonomous self-defense mechanisms that synergize with specialized innate immune cells. In addition to innate immunity, vertebrates have developed adaptive immunity (8). While innate immunity provides the first line of defense against infections or damaged cells, adaptive immunity develops at a later stage and requires the activation of lymphocytes. The innate immune system recognizes molecular structures (non-self) that are absent on the host but produced by foreign pathogens. Known as pathogen-associated molecular patterns (PAMPs), these are structures that are distinctive for the particular pathogen and include proteins, lipids, carbohydrates, and nucleic acids that are unique to the viral or microbial pathogens. Examples of nucleic acid PAMPs include single-stranded (ss) or double-stranded (ds) RNAs present in replicating viruses and unmethylated CpG DNA typical for viruses, bacteria, and fungi (9, 10). PAMPs are recognized through their interactions with a diverse set of pattern recognition receptors (PRRs) expressed by host cells. In addition to PAMPs, PRRs can recognize so called damage-associated molecular patterns (DAMPs) of endogenous origin, which are molecules released from damaged or dying cells (11). PRRs are present in most cell types, but their expression is highly abundant in certain myeloid sentinel cells such as macrophages and dendritic cells. Examples of PRRs recognizing foreign nucleic acids include: (i) cytosolic RIG-I-like receptors (RLRs), which recognize foreign RNA; (ii) Toll-like receptors (TLRs), which are transmembrane proteins in the plasma and endosomal membranes that identify “non-self” RNA and unmethylated CpG DNA; (iii) the nucleotide oligomerization domain containing (NOD)-like receptor (NLR) family pyrin domain containing 1 (NLRP1) receptor, which forms part of a macromolecular inflammasome complex; and (iv) cytosolic DNA sensors (CDSs), which detect bacterial and viral DNA (7). These pathways (briefly described below) are not mutually exclusive and can be activated simultaneously and even synergistically within the same cell.

Within the endosome, TLR3, TLR7, TLR8, and TLR9 detect foreign nucleic acids. TLR3 is responsible for detecting dsRNA and induces downstream activation of NF-κB (12, 13). DsRNA is produced by most viruses during their replication process (14). TLR7 is responsible for the detection of ssRNA. This is required by the immune system for detection of RNA viruses, especially influenza, which sequesters its double stranded RNA (15). TLR7 recognizes ssRNA sequences containing successive uridines relative to sequences with single uridines (16). TLR8 is phylogenetically and structurally similar to TLR7 and is also responsible for the detection of ssRNA. However, the localization and cytokine induction profiles for TLR7 and TLR8 differ slightly. TLR7 is predominantly expressed in the lungs, spleen, and placenta and induces IFNα and IFN-regulated cytokine production. In contrast, TLR8 is expressed in lungs and monocytes and induces predominantly TNF production (17–19). TLR9 detects non-methylated CpG-motifs found in bacterial or viral DNA (20). All nucleic acid specific TLRs, activate the adapter protein, MyD88 (21, 22), except for TLR3 that activates TRIF (23). RIG-I, MDA5, and LGP2 are categorized as RIG-I-like receptors. These receptors are involved in the sensing of RNA viruses and initiate/modulate the immune response upon virus detection (24). ()A critical component of innate cellular defense, located predominantly in cytoplasm, is RIG-I, which can differentiate foreign RNAs from native forms. The prevailing opinion is that the triphosphate on the 5’- blunt end (5’-ppp) of RNA duplexes that are at least 10 nucleotides long is required for effective recognition by RIG-I, but apparently single ssRNAs with 5’-ppp may also lead to RIG-I mediated responses as shown by its activation during influenza A virus infections (25, 26). Also, it has been demonstrated that the RNA aptamer Cl9, that is specific to RIG-I, can trigger downstream signaling in a 5’-ppp independent manner (27). The stimulation of RIG-I downstream signaling subsequently leads to production of type I IFNs and IFN-stimulated genes (ISGs) that are important for the induction of adaptive immune responses.

Importantly, the cytosolic presence of 5’-ppp dsRNA is not limited to RNA virus infection but can arise following infection with several DNA viruses and intracellular bacteria due to the transcriptional activity of DNA-dependent RNA polymerase III (RNA Pol III) (28, 29). Cytosolic RNA Pol III therefore represents an important component in host defenses against disparate intracellular pathogens. In contrast, nuclear RNA Pol III, which can transcribe a plethora of ncRNAs with diverse roles including the control of immune functions (as extensively reviewed elsewhere (30, 31), synthesizes nucleus-specific ncRNAs containing 5’-ppp that are not recognized by RIG-I under normal physiological conditions. In this case, the presence of the nuclear envelope appears to help to isolate the RNA Pol III transcripts from cytosolic RIG-I until these ncRNAs are processed further and become immunoquiescent. The largest pool of such RNA Pol III transcripts are tRNAs that are dephosphorylated by dual-specificity phosphatase 11 (DUSP11). Another strategy to avoid RIG-I recognition is to shield the 5’-ppp by binding to a protein. An example of this is the binding of the RNA component of the signal recognition particle 7SL1 (RN7SL1) with the protein signal recognition particle (SRP) (32).

**Figure 1:**
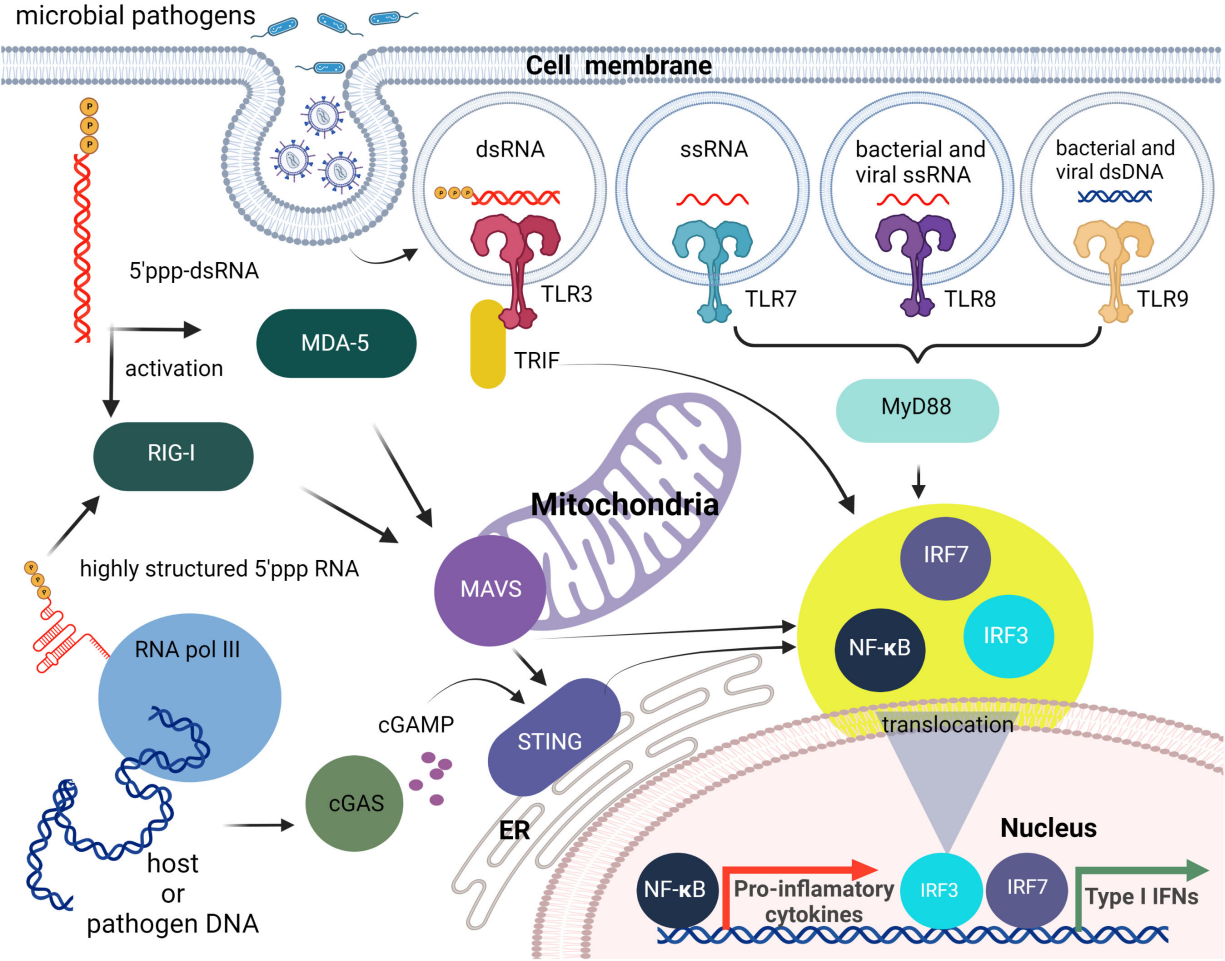
Brief overview of cellular innate immunity with an emphasis on nucleic acid recognition. The first line of nucleic acid PRRs consists of TLRs that can sense different PAMPs specific for non-self nucleic acids. Then cytosolic pathogen-associated nucleic acids can be sensed by members of the RLR family (RIG-I, MDA5). The endogenous and viral DNAs can also lead to RIG-I activation following their transcription by cytosolic RNA pol III, or can be detected directly as dsDNAs *via* cytosolic DNA sensing systems such as the cGAS-cGAMP-STING pathway. All of these pathways initiate the translocation of transcription factors including IRF3/7 and NF-κB to the nucleus and the subsequent induction type I IFN and pro-inflammatory cytokine production.

RLRs and TLR3 recognition of foreign nucleic acids converge on pathways that activate the transcription factors, interferon (IFN)-regulatory factor (IRF) 3 and IRF7, and NF-κB. IRF3/7 stimulate production of type I IFNs, whereas NF-κB induces the expression of proinflammatory cytokines, chemokines, and adhesion and costimulatory molecules that induce acute inflammation and initiate adaptive immune responses. Furthermore, crosstalk occurs between these receptors and their signaling components resulting in complex immune responses to particular viral and nonviral nucleic acids (33–36) (**Figure 1**).

The cyclic guanosine monophosphate–adenosine monophosphate synthase (cGAS) -stimulator of IFN genes (STING) pathway is an important mechanism underlying cytosolic dsDNA-induced type I IFN responses. Activated cGAS generates the signaling molecule cyclic GMP-AMP (cGAMP), which binds to STING and triggers its translocation from the endoplasmic reticulum to the Golgi apparatus. STING then activates the TBK1 kinase that, in turn, activates IRF3, leading to type I IFN gene expression. STING also responds to other cytosolic DNA including DNA-dependent activator of IFN-regulatory factors (DAI; also known as Z-DNA binding protein 1 (ZBP1)) and IFN inducible protein 16 (IFI16) (37). In addition to inducing IFN production, STING also stimulates autophagy that serves both an innate immune function by delivering cytosolic microbes to the lysosome for elimination (38), and a role in adaptive immunity as a mechanism whereby microbial antigenic epitopes are generated in the lysosomes for presentation to lymphocytes (39).

In summary, activation of PRRs in addition to other pathways, such as global inhibition of protein synthesis mediated by protein kinase R (PKR) and oligoadenylate synthases (OASes) described elsewhere, elicit multiple cellular responses including immediate host responses such as inflammation and more specific subsequent adaptive immunity that are capable of pathogen clearance and long-term protection against reinfection (40).

## Therapeutic nucleic acids and PRR agonists as immunomodulators

The presence of an intricate array of PRRs for non-self or abnormal RNA and DNA raises the safety concerns for broader applications of therapeutic nucleic acids (TNAs). Accordingly, the development of nanoparticle-carrier formulations that are immunoquiescent has obvious benefits for the translation of this highly promising biotechnology to the clinic, as severe complications, including severe inflammatory reactions that include cytokine storms and complement activation-related pseudoallergies (CARPA), are circumvented.

As recently discussed at length, the presence of an array of cytosolic and endosomal nucleic acid sensors by most mammalian cells represents a highly attractive target to bolster beneficial host immune responses to infectious agents or to augment vaccine efficacy (41). This is illustrated by the promise of nucleic acid sensor agonists such as the TLR7 agonist, imiquimod, that has been approved for the treatment of genital warts (HPV), and the recent “shock and kill” strategies aimed at eradicating latent HIV viral reservoirs using TLR7 and TLR9 ligands, such as GS-9620 (vesatolimod) and MGN1703 (lefitolimod), respectively, to initiate viral reactivation and promote immune-mediated killing of infected cells (42–44).

Efficacious vaccines require the use of adjuvants that target pattern recognition receptors on antigen presenting cells to promote their ability to deliver antigen to B and T cells, and to provide essential co-stimulation, to achieve potent and long-lasting antigen-specific humoral and cellular immune responses. Currently, there are only a handful of vaccine adjuvants that are approved for human use and most of these have limitations, such as the inability of alum to promote cellular immune responses (45). As discussed previously (41, 46), nucleic acid sensors, including TLRs, RLRs and the cGAS-STING pathway, have been an attractive target for adjuvant development. The well-known adjuvant alum is now recognized to function through the induction of endogenous DAMPs, including DNA-based TNAs, that activate nucleic acid sensing pathways (47, 48). Similarly, more recent preclinical studies have showed that modified CpG-based adjuvants or combination adjuvants, such as AS15 and K3 CpG + cGAMP, are potent inducers of both humoral and cellular immune responses, and agonists of TLR3 and MDA5, such as synthetic dsRNA TNAs including poly-IC, the RNase-resistant derivative poly-ICLC (Hiltonol), and poly-IC12U (Ampligen), have been explored for clinical use (46, 49, 50). Furthermore, the TLR7 and TLR8 agonist 3M-052, formulated in a lipid-based nanoparticle (3M-052-AF), is being evaluated as an adjuvant for a preventive HIV vaccine, while a liposome formulated cyclic dinucleotide-based adjuvant has been shown to protect against a range of influenza strains (51, 52). As such, the array of nucleic acid sensors expressed by mammalian cells, as well as the identification of natural and synthetic ligands for these receptors, represents tremendous potential for the development of novel and effective adjuvants

## Endogenous noncoding RNAs as immunomodulators

RNA provides diverse functions; classically, RNA allows for the flow of genetic information from DNA to proteins by mRNA translation, where tRNA and rRNA are prominent in facilitating expression with the help of post-transcriptional regulation *via* RNAi. The other noncoding (nc) RNAs participate in splicing and, thus, finalize the functional mRNA sequence. Besides this, a diverse cornucopia of short or long ncRNAs are involved in physiological as well as pathological processes, often described with little detailed mechanistic understanding. Most interactions are carried out in association with proteins and all processes are spatially and temporally controlled, which allows sensing of potentially pathogenic conditions and the alerting of host defensive systems (53). Hence, a better understanding of these processes and the ncRNAs involved may identify new targets for therapeutic intervention.

While a detailed understanding of the exact physiological roles of endogenous ncRNAs in innate system are only now emerging, it has become clear that dysregulation of their transcription, processing, and trafficking can have serious impact on RIG-I activation. Similarly, the participation of endogenous ncRNAs is open for therapeutic exploitation, either as a target or an effector, and their potential has recently been explored for some RNA Pol III transcripts (32).

The development and use of immune checkpoint inhibitors that disrupt co-inhibitory T-cell signaling has revolutionized cancer therapy. Upon relieving such blockade, the most efficient T-cell anti-tumor responses occur in an inflammatory microenvironment where there is an increased expression of type I IFNs, ISGs, pro-apoptotic molecules, and T-cell attracting chemokines. Many therapeutic strategies have focused on inducing inflammation within tumors and an attractive emerging strategy has been to exploit cellular nucleic acid PRRs (54).

Furthermore, the controlled stimulation of RIG-I in cancer cells using ligands that mimic an infection represents a new adjunctive therapeutic approach by increasing the susceptibility of tumor cells to conventional treatments. Such a possibility is supported by the observation that patients with intact RIG-I signaling are responsive to radio- and chemotherapy, while those with RIG-I suppression show tumor resistance (55). In addition, RIG-I activation renders cultured cancer cells susceptible to natural killer cell-mediated killing and promoted phagocytosis of tumor cells *in vivo* (56, 57). The intrinsic molecular heterogeneity of tumor cells within each patient generally requires a combinatorial approach. For example, the simultaneous suppression of tumor cell survival by targeting factors such as Bcl-2 or TGF-ß using RNAi approaches while simultaneously increasing the immunogenicity of tumor cells by activating RIG-I with 5’-ppp RNAs can decrease tumor viability. In summary, the combination of traditional approaches with emerging immunostimulatory treatments holds the promise of improving clinical outcomes (58–62).

**Figure 2:**
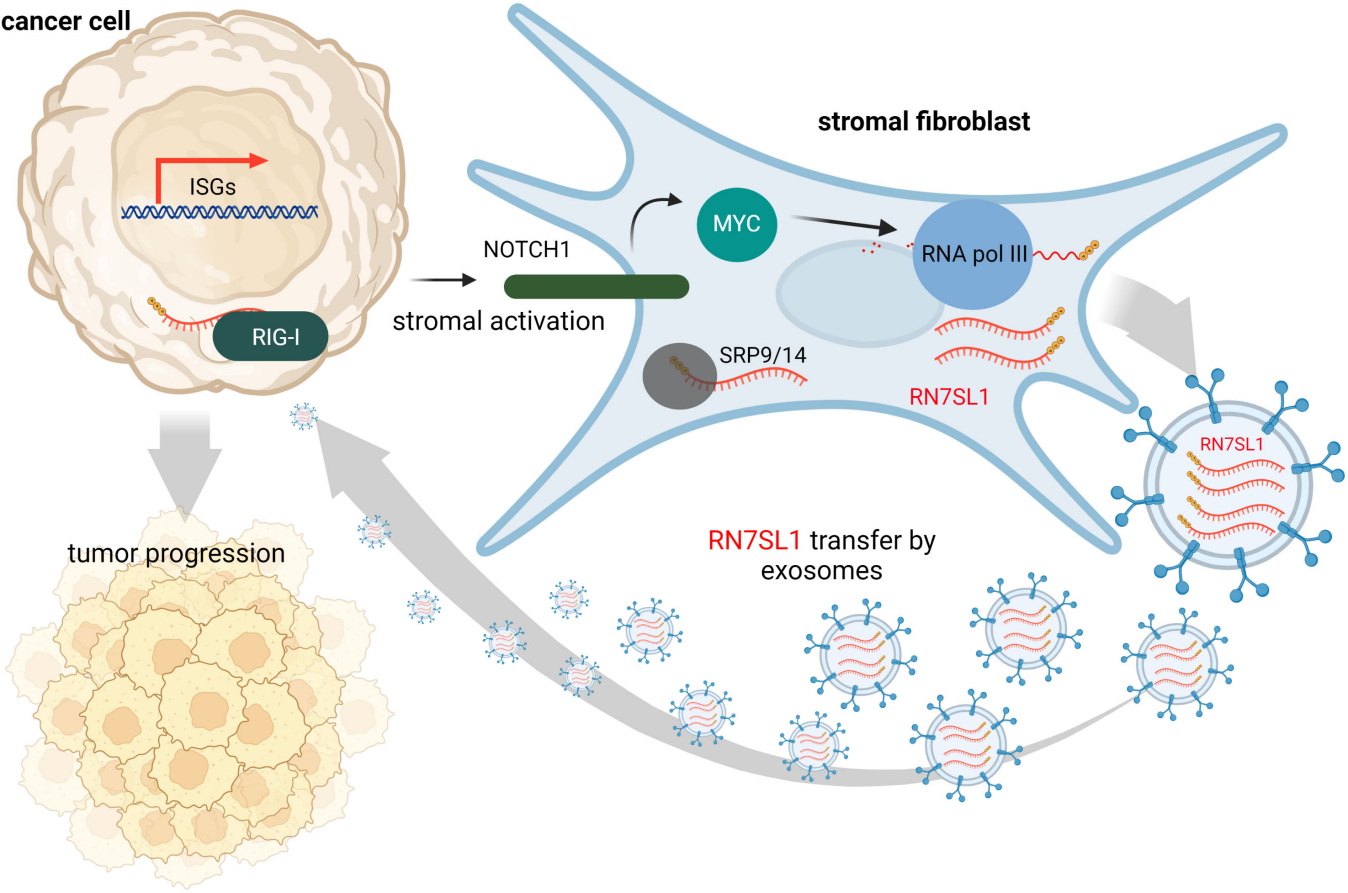
During contact with stromal fibroblasts, breast cancer cells activate NOTCH1/MYC signaling that leads to higher transcription of ncRNA RN7SL1 carrying 5’-ppp. These transcripts then remain unshielded since levels of their protein-binding partner (SRP9/14) remain constant. Naked RN7SL1 is loaded to exosomes and, upon interaction with breast cancer cells, can activate RIG-I signaling leading to an inflammatory tumor microenvironment that can promote tumor progression and poor clinical outcomes.

Contrary to this, many tumors express high levels of ISGs in response to DAMPs and inflammation at the tumor site is often associated with cancer progression and treatment resistance. It is likely that, under various stress conditions induced by cancer treatment, endogenous RNAs can serve as DAMPs *via* as yet poorly understood mechanisms. Under physiological conditions, epithelial cells are typically not in contact with fibroblasts, but they may interact at wound sites or at sites of tumor invasion. Such tumor-stromal cell interactions may then lead to damage signal release that could prove crucial for tumor invasiveness and resistance to therapy (63). Emerging evidence suggests that ISG activation in responsive tumor cells (e.g., breast cancer) by specific ncRNAs from stromal cells promotes survival and progression of cancer (32). Exosomes that deliver RN7SL1 ncRNA generated by RNA Pol III were identified as the pivotal link between activated stromal cells and RIG-I dependent activation of ISG signaling in breast cancer cells. While RN7SL1 is shielded by SRP9 and SRP14 to avoid detection by RIG-I under normal circumstances, naked RN7SL1 is transferred to stromal exosomes following contact between fibroblasts and ISG-R breast cancer cells. The unshielding of RN7SL1 and its loading into exosomes is a consequence of a disrupted stoichiometry between RNA Pol III-driven transcription and unchanged SRP expression. This imbalance is induced by stromal NOTCH1-MYC signaling which, in turn, is enhanced by contact-dependent signaling by breast cancer cells. As a result, RN7SL1 delivered by exosomes to breast cancer cells activates RIG-I signaling (**Figure 2**) (32).

The immune recognition of RN7SL1 ncRNA has been employed in a follow up study where it was used to enhance the function of chimeric antigen receptor T-cells (CAR-T cells) (64). CAR therapy has recently emerged as a major advance in cancer immunotherapy with six different CAR-T cell products having been approved by the US Food and Drug Administration thus far. This treatment is based on T-cells isolated from the patient’s body and customized to their needs by genetic engineering to express recombinant chimeric antigen receptor (CAR) proteins on their membranes. CAR-T cells are then expanded ex vivo and introduced into the patient where they continue to divide and, using the engineered receptor, identify and eliminate cancer cells displaying the specific antigen. CAR-T therapy has shown remarkable efficiency in some hematologic cancers, but application of this treatment to solid tumors has remained challenging. Poor infiltration of CAR-T cells into the tumor microenvironment, immunosuppressive conditions at the tumor site, and poor expansion of CAR-T cells, are some of the issues that may be responsible for these problems

**Figure 3:**
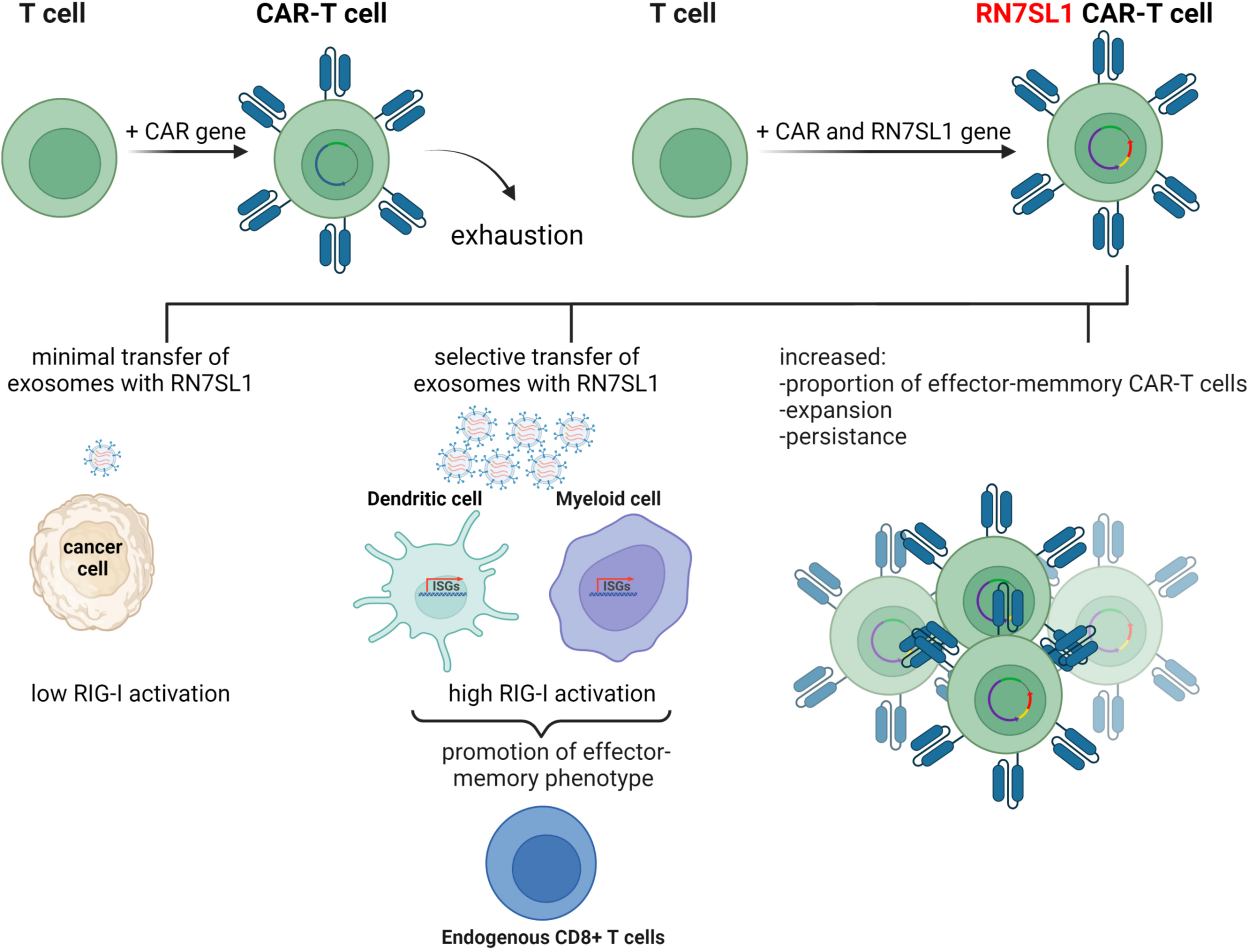
The use of endogenous RN7SL1 ncRNA to improve CAR-T cell therapy efficacy. Engineered CAR-T cells transcribe transgenic RN7SL1 ncRNA together with a chimeric antigen receptor. The resulting cell-autonomous effect prevents T-cell exhaustion and increases cell expansion. In addition, excreted exosomes transport RN7SL1 to intratumor myeloid cells, such as dendritic cells, rather than cancer cells, thereby avoiding inflammation triggered by tumor cells.

To improve the performance of CAR-T cells in such solid tumors, a plasmid encoding ncRNA RN7SL1 was used as a key component in an experimental treatment. RN7SL1 ncRNA was overexpressed in CAR-T cells and was found to activate IFN production in murine and human immune cells. A construct expressing two clinically relevant CARs, the M5BBz CAR targeting human mesothelin (MSLN) and the 19BBz CAR against human CD19, and RN7SL1 driven by the U6 promoter was then developed and tested. It was found that most of the expanded CAR-T cell population that expressed RN7SL1 RNA showed a memory T-cell phenotype and persisted longer in both the tumor and the bloodstream than RN7SL negative CAR-T cells, which were quickly exhausted. RN7SL overexpression resulted in its translocation to exosomes and its predominant export to immune cells residing in the tumor microenvironment, but not cancer cells, leading to IFN signaling. This, therefore, prevented the immunosuppression and tumor progression previously observed in another study (**Figure 3**) (64). Since RN7SL1 ncRNA was transcribed from an engineered construct here, it raises the intriguing question of whether natural and synthetic 5’-ppp ncRNAs provide similar immunostimulatory activity in such a system.

Interestingly, a role for a long ncRNA (lncRNA) in RIG-I regulation has been observed in a murine virus infection model. The endogenous lncRNA, lnc-Lsm3b, is normally present in the cytoplasm at low copy numbers, but such expression was increased tenfold after infection with Sendai virus or vesicular stomatitis virus (VSV). This upregulation was shown to be induced by a high concentrations of type I IFNs in a time dependent manner. Surprisingly, lnc-Lsm3b transcription silencing during infection resulted in higher type-I IFN production, which suggests that lnc-Lsm3b may suppress RIG-I activation at late stages of infection (65). While the therapeutic potential of lnc-Lsm3b binding motifs as RIG-I decoys is obvious, it remains unclear whether such treatments would be similarly effective in decreasing of RIG-I activity in human subjects.

## Aptamers as extracellular immunomodulators

Aptamers are single-stranded nucleic acids (RNA, DNA, or chemical analogs) selected to adopt a conformation that allows for the highest binding affinity and specificity to its pre-defined target. The correct aptamer sequences are identified during a process called SELEX (systematic evolution of ligands by exponential enrichment), where the library of ~10^12^ different oligonucleotides is presented to the target molecule and subjected to several rounds of selection (66). Due to their known sequence and batch-to-batch consistency, aptamers selected against certain receptors can be used similarly to their monoclonal antibody (mAbs) analogs to either prevent receptor interactions with its natural ligand or inhibit/activate receptor downstream signaling. However, compared to mAbs, aptamers have a greater shelf life, and their storage and transportation does not require cold chain maintenance. Also, aptamers have additional benefits as they are synthetic and can be manufactured in significantly less time than mAbs, as the chemical synthesis of aptamers does not require living systems. Furthermore, aptamers are amenable to chemical modifications and precise conjugation to other drugs and imaging agents.

Cell-to-cell interactions between cancer and immune cells represent a crucial interplay for tumor survival. One aspect of this communication is represented by immune checkpoints, receptor-ligand pairs expressed on the cell surface that control the strength of T-cell activation under physiological conditions. When T-cells recognize checkpoint proteins on tumor cells that are often overexpressed, it sends an inhibitory signal that prevents T-cell attack. Therefore, aptamers with proteins involved in the inactivation of co-immunostimulatory pathways on the one side and activators of signaling that lead to immune quiescence on the other represent potent prospective therapeutic agents. Given that the concept of immune checkpoint inhibitors has revolutionized cancer immunotherapy, it is not surprisingly that several monoclonal antibodies targeting such interactions have already been approved and many clinical trials are ongoing (67). Furthermore, it is fully appreciated that the use of antibody combinations against multiple targets can exert synergistic effects.

**Figure 4:**
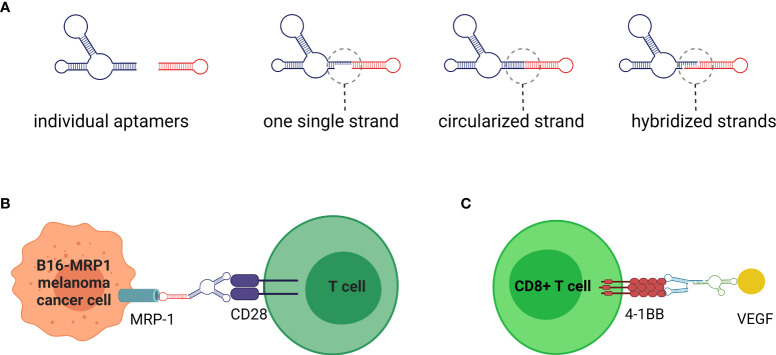
Aptamers are nucleic acids selected to specifically bind the molecules of interest in a similar manner to monoclonal antibodies. **(A)** Nanotechnology offers significant advantages in fusing individual aptamers to multivalent or bispecific molecules. Thus, by linking together the same or different aptamers, the increased binding affinity and/or ability to crosslink target cell receptors can be achieved. **(B)** Bispecific aptamers can promote cell-to-cell interactions with potential immunomodulatory applications. For example, a single stranded bispecific aptamer targeting CD28 on T cells and Multidrug-Resistant-associated Protein 1 (MRP1), involved in chemotherapy on B16 melanoma cancer cells, has been used to provide the necessary co-stimulatory signal for T cell activation. **(C)** Instead of cell membrane receptors that may be quickly internalized, an alternative strategy could be to target co-stimulatory signals to proteins (e.g., VEGF) overexpressed on tumor stroma.

The application of extracellular immunomodulating aptamers has contributed to this therapeutic approach with several original concepts, as extensively reviewed by Thomas et al. (68). Due to the programmability of nucleic acids, aptamers can be rationally designed to assemble into higher order structures that enhance or even alter their original functionality. Such a relatively simple approach cannot be achieved with mAbs. The multivalent, and usually bispecific, aptamers can be designed and synthesized as a single continuous sequence, hybridized, or circularized (**Figure 4A**). The combinatorial potential of linking aptamers together offers not only the possibility of creating multivalent aptamers targeting the same or different epitopes of the same target molecule, but also the assembly of aptamers targeting diverse proteins. This presents an opportunity to promote specific cell-to-cell interactions, where the immune cell can anchor to the tumor cell and provide co-stimulatory signals more efficiently (**Figure 4B**) (68–71).

Alternatively, before T-cell interactions with cancer cells, the co-stimulatory signal on T-cells could be triggered by a bispecific aptamer targeted to abundant protein, such as VEGF that is secreted to the tumor stroma and linked to an agonistic aptamer specific for an inducible costimulatory receptor, such as 4-1BB. This approach has been tested in a murine model and was found to outperform the administration of an agonistic 4-1BB Ab or 4-1BB aptamer alone (**Figure 4C**) (72).

One of the first studies to explore the binding of antagonist RNA aptamers to T-cells expressing the negative co-stimulatory molecule CTLA-4 showed that integration of four individual aptamers into a tetravalent structure increased its bioactivity in a murine model (73). Similarly, linking two RNA aptamers targeting the co-stimulatory aptamer, exerted co-stimulatory activity on cytotoxic CD8+ T cells *in vitro* and promoted tumor rejection *in vivo* (74). An interesting functional change was described in two 2’-F modified RNA aptamers specific for the CD28 receptor for B7. Binding of one aptamer prevented co-stimulation *via* CD28, while binding of a second aptamer did not have a functional outcome. However, when both aptamers were linked together, either by double-strand linker or fusion into a single-strand molecule, their binding led to CD28-mediated activation (75). Gain of function upon assembly of aptamers on the scaffold was also observed in the T cell costimulatory receptor of T cells, which, in the monomer state, does not stimulate OX40. However, the annealing of two RNA aptamers to two separate complementary DNA oligonucleotides, linked by a polyethylene spacer, led to dimerization of OX40 and subsequent activation of downstream signaling (76). Indeed, the linking of individual aptamers in one complex represents a potentially versatile combinatorial therapeutic tool.

Although the principle of agonistic or antagonistic aptamers in immunomodulation is relatively straightforward, many technological and biological challenges remain. The engagement of aptamers with cell surface molecules implies their delivery in a naked form, which exposes them to degradation by serum nucleases. Traditionally, replacing natural nucleotides with chemical analogs, either during the SELEX process or post-selection, increases nucleic acid resistance to nucleases (77). After therapeutic application, aptamers, due to their small size, have a high chance of penetrating the tumor microenvironment. However, their small size negatively affects the rate of clearance, which is a contributor to half-life in the blood. To overcome their shorter half-life *in vivo*, a higher dose of aptamers might be required to increase their duration in the blood and allow for delivery to target tissues and cells.

The choice of target also determines the functional output. To prevent side effects, the selected target receptor molecule should ideally be as tumor cell specific as possible. Furthermore, receptor turnover rate is an important factor that affects the effectiveness of bispecific aptamers mediating cell-cell interactions. Rapid internalization with bound aptamer decreases the chance of establishing physical interactions between the cells. In other words, the receptor has to be displayed on the surface for sufficient time to allow the creation of a synapse between the immune and tumor cells.

## Nucleic acid nanoparticles as intracellular modulators

**Figure 5:**
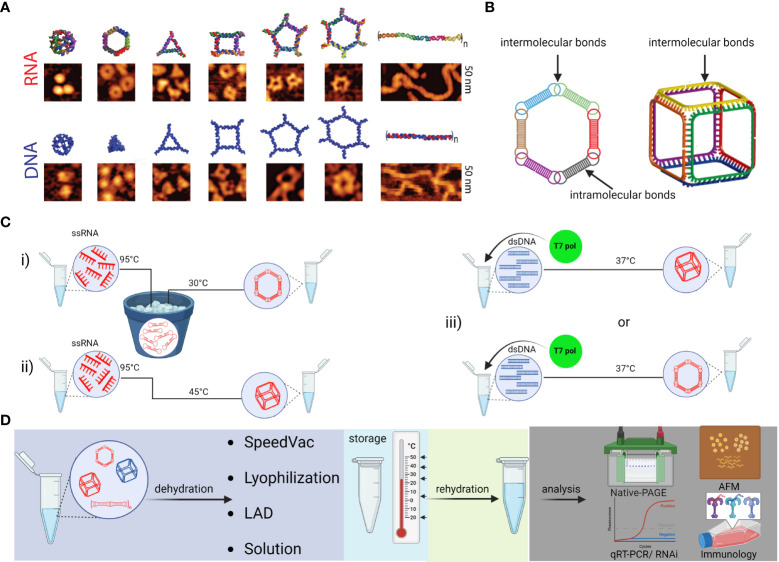
Schematic depiction of various NANPs, their production, characterization, storage, and handling. **(A)** Computational 3D visualization of individual NANPs with corresponding representative AFM images. **(B)** Two orthogonal NANPs design strategies are based either on the presence of both intra- and intermolecular or only intermolecular bonds, which also determine the assembly protocol of corresponding NANPs. **(C)** Several protocols for efficient one-pot NANPs self-assembly. Protocol (i) promotes secondary structure formation of individual monomers needed for NANPs assembly *via* long-range interacting motifs. For this assembly protocol, the individual ssRNAs are first denatured by heating at 95°C and then snap cooled on ice to form intramolecular Watson-Crick (W-C) bonds. The following incubation at 30°C in the presence of Mg^2+^ ions allows intermolecular bindings of monomers and assembly of NANPs. In (ii)> protocol, monomers form only intermolecular canonical Watson-Crick base pairs, thus no pre-folding is needed, and any intramolecular interactions should be avoided by design. The (iii) protocol allows for co-transcriptional assembly of different types of NANPs formed as their RNA strands are transcribed from dsDNA templates. **(D)** Assembled NANPs can be stored and transported in anhydrous forms at ambient temperatures. The impact on structure stability, immunorecognition, and functionality depends of dehydration protocol and needs to be checked after rehydration for each type of NANP.

NANPs are innovative scaffolds composed of rationally designed oligonucleotides or oligonucleotide chemical analogs. Because of their biocompatibility, functional versatility of nucleic acids, and tunability of their physicochemical and biological properties, NANPs have demonstrated strong potential for the development of future nanomedicine. Both RNA and DNA can form intra- or intermolecular hydrogen bonds *via* canonical base pairing, allowing for design and assembly of an almost limitless library of architecturally diverse nanoscaffolds with high batch to batch consistency (78, 79). The presence of a 2’-OH group in RNA ribose sugars enables RNA to adopt more sophisticated geometric optimization which expands the repertoire of possible hydrogen bonds classified in 12 geometric families (79–81). This is why RNA molecules naturally present a plethora of structural and long-range interacting motifs that can be engineered into NANPs with precisely controlled shapes (*e.g.*, 3D vs 2D vs 1D), sizes (10-100 nm), and compositions (RNA *vs* DNA *vs* chemical analogs); various functionalities and bioactive properties can be encoded in the NANPs’ architectures (82–85) (**Figures 5A, B**). Functionalization of NANPs can be achieved *via* self-assembly of different TNAs, either using toeholds or by incorporating TNAs directly into the sequences of nanoscaffolds. Both approaches allow the same NANP scaffolds to be formulated with different TNAs and other functionalities. For example, hexameric RNA rings have been designed to carry multiple aptamers (*e.g.*, specific for human epidermal growth factor receptor), siRNAs targeting various genes, and fluorophores for NANPs’ visualization in cells and *in vivo* (82).

The psychochemical properties of NANPs are favorable for the pharmaceutical industry. Depending on the overall design principles and composition, various NANPs can be assembled under several simple protocols (**Figure 5C**). Assembled NANPs can be subsequently stored and transported in solution on ice or can be dehydrated and handled at ambient temperatures. In a recent study, several novel protocols for drying NANPs were compared with traditional lyophilization methods (86). It was discovered that while the light assisted drying (LAD) approach was fine-tunable and more reproducible in retention of NANP structures upon rehydration, this approach only allowed for processing a relatively small volume of NANPs solution, and processing of only one sample at the time. Lyophilization permits high throughput processing while also preserving structural stability of NANPs, but the retention of biological functionality becomes questionable. Addition of cryoprotectants such as trehalose seemed to aid in reducing the potential structural damage, but more investigation is necessary to reveal biological and immunomodulatory potential of trehalose preserved NANPs in clinical settings.

The physiochemical properties of nucleic acids also affect their immunostimulatory properties. Unsurprisingly, the immunorecognition of NANPs is dependent on nucleic acid composition, size, and dimensionality as PRRs recognize distinct ligand motifs (87–89). Using these parameters as predictive indicators of immunostimulatory properties, NANPs can be designed to either be immunoquiescent or enhance desired immunological responses. For example, the NANPs composed of DNA are consistently immunoquiescent when transfected into human immune cells but this was not the case for their RNA analogs (88). The proportion of DNA and RNA can be specified during construction of DNA/RNA hybrid NANPs for the desired immune response or lack thereof. Similarly, the vast library of planar and globular NANP shapes allows for further optimization of this immunomodulator scaffold. Globular 3D NANPs made of RNA induce the strongest immunorecognition while the fibrous 1D NANPs are the least immunostimulatory (90). Furthermore, incorporation of modified nucleic acids can be utilized to avoid certain recognitions *via* specific PRRs. For example, incorporation of 2’-fluoro modified pyrimidines in NANP strands abrogate TLR7-dependent immune responses (91).

NANPs complexed with TNAs display great promise as immune response modulators. Importantly, the NANP scaffold allows for controlled and coordinated delivery of multiple functional groups to the same cell (92). For example, the individual strands of NANPs functionalized with a combination of different TNAs and TNA-functionalized RNA ring nanoscaffolds that target all four variants of lysophosphatidylcholine acyltransferases (LPCATs) significantly increased susceptibility of melanoma cells to radiation treatment (93). Notably, recent evidence indicates that the orientation of added TNAs can additionally contribute to the immunostimulatory properties of NANPs. Accessibility of NANP components to PRR binding may contribute to the observed difference in their immunostimulation. For example, despite the number of 5’-ppp remaining constant for functional and non-functional NANPs, RIG-I was specifically activated in response to transfected NANPs that carried TNAs. The response for some orientations of TNAs was stronger than for others. This data indicates that the cytosolic sensor, RIG-I, can distinguish between non-functional and functional NANPs and the extent of functionalization (94).

**Figure 6:**
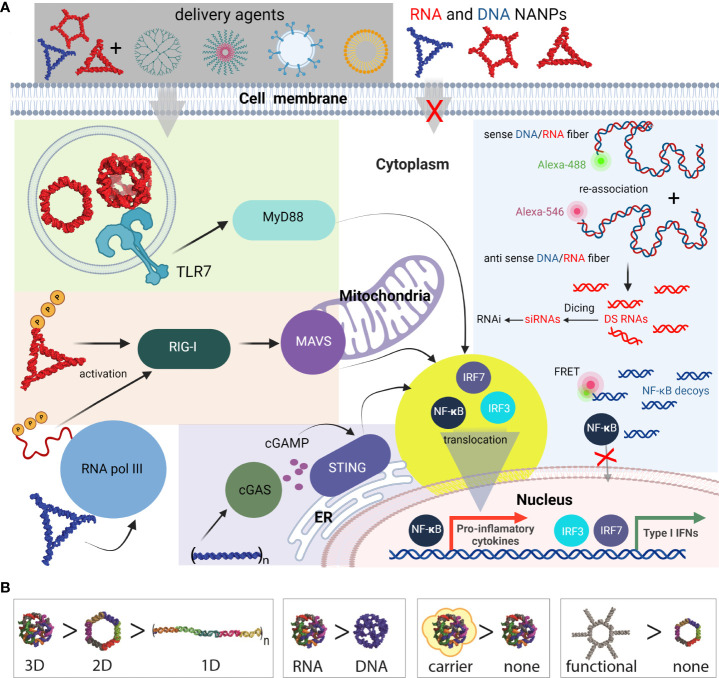
Most common innate pathways shown to be activated upon NANPs internalization. **(A)** Intracellular delivery of NANPs requires carriers; naked NANPs are immunoquiescent due to their ineffective crossing of biological membranes. Delivery of RNA rings and cubes trigger the immune system through TLR7. By-passing of TLR sensing can be compensated by RIG-I that can detect RNA NANPs bearing 5’ triphosphates. DNA containing NANPs can be sensed after promoter independent transcription of NANPs strands by RNA pol III. DNA fibers stimulate cellular immunity through cGAS-cGAMP-STING pathway. Additionally, interdependent DNA/RNA fiber NANPs can be rationally designed to release of RNAi inducers and NF-κB decoys upon their intracellular re-association. This results in gene specific silencing while simultaneously blocking NF-kB translocation to nucleus thus lowering the proinflammatory immune responses. **(B)** Some of the architectural and compositional parameters that define immunorecognition of NANPs.

Functional group delivery can be further controlled through the intracellular reassociation of RNA/DNA hybrid NANPs. In this scenario, a pair of complementary RNA/DNA hybrids are engineered to be non-functional by carrying various split RNA functionalities such as RNAi inducers and aptamers (95–98). When interdependent RNA/DNA hybrids are both present in the cytosol, there is complementary base-pairing at toehold regions that drive branch migrations and the release of functional groups (89, 95, 96). This system has been used to effectively deliver dicRNAi inducers for the knockdown of gene expression of HIV-1 and relevant oncogenes *in vitro* (83). Additionally, RNA/DNA fibers have been optimized to deliver and activate both RNAi inducers and DNA decoys, targeting NF-κB, a transcription factor that induces production of proinflammatory cytokines (**Figure 6**) (99). These NF-κB targeting NANPs display great promise for reducing inflammatory immune response, as the decoys function to prevent translocation of activated NF-κB to the nucleus. In addition, the RNAi inducers may serve to reduce overall NF-κB expression.

In summary, the immune responses elicited by functionalized NANPs depend on their shape. This has been previously shown where all RNA-made NANPs, when functionalized with TNAs on each monomer, induced high levels of type I (IFNα, IFNβ, and IFNω) and type III (IFNλ) IFN responses. IFN responses to RNA cubes, rings, and fibers where every monomer was functionalized were comparable to ODN2216, a CpG oligonucleotide, a known IFN inducer. However, when the fibers were only functionalized on every other monomer, IFN response were significantly decreased, indicating that the spacing between the functionalization groups plays an important role in PRR activation. This decrease in IFN responses was mirrored by decreases in the proinflammatory responses evoked by the NANPs with the same amount of siRNA delivered (83).

## Combinations of different carriers and NANPs as intracellular modulators

In the absence of a carrier, the negative charge of all of our NANPs prevented penetration through biological membranes (88, 100). As such, a variety of carriers, including cationic lipids, liposomes, polymers, magnetic nanoparticles, mesoporous silica-based nanoparticles, and exosomes, most be employed as agents to protect against nuclease degradation and to facilitate delivery (91, 100–103). Furthermore, in the absence of a carrier, NANPs are essentially invisible to cells and so are immunoquiescent (104–106). This makes them perfect candidates for extracellular use (107). Cationic lipids and liposomes have been extensively explored as carriers for TNAs and can also serve as viable carrier options for NANPs. The transfection reagents lipofectamine and DOTAP have been previously employed to deliver NANPs to non-immune and immune cells (106, 108, 109). However, the use of Lipofectamine 2000 as a carrier is limited to *in vitro* cell delivery. Alternatively, cationic bolaamphiplies form highly stable delivery vesicles that can be utilized *in vitro* and *in vivo* due to low toxicity. Notably, bolaamphiplies are a promising carrier as previous studies indicate that they deliver siRNAs across biological barriers, including the blood brain barrier (110, 111). Similar to lipid-based carriers, polymers, such as polyethylenimine (PEI), poly(β-amino esters), polyamidoamine (PAMAM) dendrimers, and branched PEI, can be employed to deliver TNAs (100, 101, 112). The cationic, amphiphilic co-polymer, poly(lactide-co-glycolide)-grafted-polyethylenimine (PgP) is a micelle forming co-polymer that can deliver both TNAs and TNA functionalized NANPs to multiple cell types *in vitro* (100) (95). Recent data also indicates that PgP effectively delivers functional NANPs following retro-orbital administration in mice models. Similar to PgP, due to electrostatic interactions, NANPs can be complexed with cationic PAMAM dendrimers (101). These dendrimers facilitate NANP uptake to adherent cell lines and PBMCs. Finally, exosomes, 30 – 150 nm vesicles released upon fusion of multivesicular bodies with the cell membrane, mirror the characteristics of the parent cell. Exosomes facilitate cellular communication as cargo is delivered to neighboring cells by either receptor-mediated endocytosis, micropinocytosis, or membrane fusion. Exosomes have been documented to effectively deliver TNAs and functional NANPs *in vitro* and in murine *in vivo* models to target cells and/or tissues (113, 114). Exosomes also defend against nuclease degradation and efficiently deliver NANPs of differing three dimensional conformations functionalized with siRNA.

Importantly, carrier selection affects NANP immunostimulatory properties. First, the carriers discussed above can have immunostimulatory properties independent of the NANPs. Additionally, carrier selection determines both the efficiency of NANP delivery to specific cell types and the cellular route of NANP entry (91). NANPs complexed with lipid-based carriers have been demonstrated to first traffic through an endosomal compartment prior to delivery to the cytosol (88, 91). RNA cubes, rings, and fibers stimulate varying degrees of proinflammatory and IFN responses in part due to recognition *via* endosomal TLRs. In contrast to lipofectamine delivery, using a cationic amphiphilic co-polymer carrier stimulates reduced inflammatory cytokine production and no IFN production (100) Likewise, NANPs delivered with dendrimers are largely immunoquiescent (101).

Due to highly cell type specific expression and subcellular localization of PRRs, carrier selection can also impact nucleic acid sensor detection of NANPs thereby altering the subsequent immune responses. Previous studies using human peripheral blood mononuclear cells have indicated that plasmacytoid dendritic cells are the primary producers of IFNs following delivery of NANPs complexed with a lipid-based carrier (88, 91, 106). Notably, this observation supports results in reporter cell-lines indicating that RNA cubes and rings activate TLR7 and TLR9 as plasmacytoid dendritic cells are known to express the endosomal Toll-like receptors, TLR7 and TLR9.

NANPs can be designed to have switchable, tunable, and programable properties for a number of applications. As noted above, RNA cubes are the most immunostimulatory. While similar in shape, DNA and RNA cubes have different immune responses for the same carriers; RNA cubes induce significant amounts of IFNα and IFNω, while DNA cubes only produce IFNβ and IFNλ. RNA- and DNA-based rings have been found to be more immunostimulatory than their fiber counterparts (88). NANPs have been designed to interact with the immune system *via* their structure (88) and used to address specific biochemical problems (99, 115, 116). This includes the use of NANPs as scaffolds to carry TNAs with controlled and tunable immunostimulants (113) and the use of functionalized NANPs to silence specific genes to inhibit virus production (82). This has been achieved through the application of our knowledge of the structure and function of natural and artificial classes of nucleic acids to NANP structure. Furthermore, known therapeutics and targeting agents can be attached to NANPs, and used for drug delivery, biosensing, and as molecular devices (89, 95, 115, 117–119).

Together, these studies indicate that cellular responses to NANPs is dependent on their structure, composition, and functionalization, in addition to type of carrier employed for intracellular delivery. Previous work has shown trends in the degree of immune response based on the previously mentioned design features (106) (101). Differences in dimensionality (1D, 2D, and 3D), composition (DNA or RNA), and connectivity (intramolecular, intermolecular, or both) evoke varying immune responses and enable NANPs to be customized based on the intended therapeutic effect (107). The field of therapeutic nucleic acids continues to advance and holds the promise of the development of versatile new means to manipulate host cell machinery to achieve a desired therapeutic effect in the absence of detrimental recipient responses (120, 121).

## Translation of immunomodulatory nucleic acid therapeutics to the clinic

The immunomodulatory nucleic acids can be divided to two groups. In the first group, nucleic acids deliver genetic information that translates to immunogenic/immunomodulatory proteins such as chimeric antigen (CAR-T therapy) or nucleic acid-based vaccines (mRNA vaccines and adenovirus delivered vaccines). The second group contains noncoding nucleic acids that directly interact with proteins involved in immune pathways.

**Figure 7:**
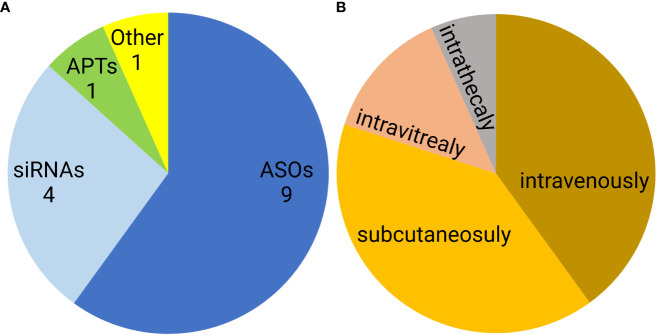
Distribution of FDA approved noncoding nucleic acid therapeutics according to type **(A)** and route of administration **(B)**. ASO- antisense oligonucleotides, siRNAs- small interfering RNAs, APTs- aptamers.

Noncoding nucleic acid-based therapeutics are only slowly entering medical use. Since 1998, when first oligonucleotide drug, Vitravene (also known as Fomivirsen) was approved by the FDA, only 15 non-coding oligonucleotides have been approved for clinical use. This group contains nine antisense oligonucleotides (ASOs), four siRNAs, one aptamer, and one natural oligonucleotide product made by depolymerization of porcine intestinal mucosal DNA (122–124) (**Figure 7A**). Six of these formulations are administered subcutaneously, another six are administered intravenously, two intravitreally, and one intrathecally (**Figure 7B**). However, none of the approved oligonucleotides are intended to be immunostimulatory. Recently, several clinical trials of a short synthetic RNA ligand that is selective for RIG-I, RGT100, have been conducted. One of these studies employing such a ligand (MK-4621) has been terminated due to business concerns (125), but this agent was found to activate RIG-I and contribute to modest antitumor activity, albeit with no substantial improvement over current treatments (125). In addition to antitumor activity, RIG-I agonists have been examined in preclinical trials as antiviral agents (126). Specifically, short hairpin RNA SLR14 complexed with polyethyleneimine was found to protect against SARS-CoV-2 infection in human angiotensin-converting enzyme 2 transgenic mice (126).

Rintatolimod is a dsRNA composed of inosinic and cytidylic acid residues that stimulates TLR3 but not cytosolic helicases. In addition, it activates 2′-5′ adenylate synthetase. First identified in the 1970s, rintatolimod has been tested clinically for the treatment of various conditions including breast and ovarian cancers and HIV infections. However, to date, the FDA has only granted it an orphan drug designation status for patients with pancreatic cancer in 2020 and the treatment of Ebola virus infection in 2022 (127). It is currently in Phase II and III double-blinded, randomized, placebo-controlled clinical trials for the treatment of chronic fatigue syndrome/myalgic encephalomyelitis (CFS/ME), and has shown promising results (128, 129).

Another group of noncoding immunomodulatory RNAs that are being tested in clinical trials include two spiegelmers, the L-stereoisomer RNA acid aptamers (130). PEGylated NOX-E36 binds the chemokine CCL2 thereby preventing the infiltration of CCR2-dependent tumor associated macrophages that initiate tumor-supporting angiogenesis (131, 132). In contrast, the NOX-A12 spiegelmer’s target is CXCL12 that is implicated in the exclusion of T cells from the tumor microenvironment, and so blocking the actions of this chemokine should lead to increased protective T-cell infiltration. Excitingly, NOX-A12 has recently been studied in patients with advance stage colorectal and pancreatic cancer where it has been shown positive synergistic effects when combined with the PD1 immune checkpoint inhibitor, Pembrolizumab (133, 134).

## Summary

Innate immunity is an evolutionary conserved network that provides immediate protection and precipitates specific and long-term adaptive immunity. In addition to providing defense against infectious organisms, the innate immune system can recognize danger signals that originate from cell stress and/or tissue injury. The integration of a wide range of signaling pathways initiated by exogenous and endogenous stimuli culminates in the expression of genes that underlie responses that include inflammation. The existence of certain types of nucleic acids and their cellular location is closely monitored by PRRs. These molecules play an important role in distinguishing foreign or altered self-nucleic acids, or their presence in appropriate locations, that can be manifestations of viral and bacterial infection or cellular damage/transformation. Due to their physicochemical properties, biocompatibility, and easy synthesis, nucleic acids may represent an ideal tool to manipulate the immune system. Immunogenic motifs from virus transcripts or RNA genomes can be derived and synthetically selected sequences or cellular ncRNAs can be employed. However, the potency of individual immunogenic ncRNAs is currently unknown and their activity is likely to differ based upon the particular application. However, the use of NANP nanotechnology alone has already identified many of the properties, such as composition, architectural parameters, dimensionality, size, and chemical stability, that define the immunogenicity of such structures (135). The complications experienced in translating simple TNAs to clinical therapies are also important considerations for complex NANPs, but it should be noted that the properties of NANPs are more than the sum of their constitutive parts (120). While resistance to nucleases and renal clearance can be solved relatively easily, other safety and efficacy concerns remain challenging. Targeted delivery, barrier penetration, and toxicity, remain the principal obstacles for nucleic acid therapeutics. This problem is compounded by the current nonexistence of FDA guidance documents for such agents, in contrast to other strategies including gene therapy. Another issue is the scalability of NANP synthesis for mass production and the present lack of simple and unified assembly protocols.

Despite these issues, it is clear that NANP technology holds great promise and has high therapeutic potential. Over the last decade, we and others have explored the possibility of multifunctional NANPs that carry diverse functional moieties (aptamers, siRNAs, ASOs, decoys, etc.). It now remains to combine immunostimulatory ncRNAs with NANP scaffolds to create new multi-tasking NANPs that permit conditional activation as the next generation of nucleic acid-based theranostics.

## Author contributions

All authors listed have made a substantial, direct, and intellectual contribution to the work and approved it for publication.
